# Pharmacoepidemiological aspects of outpatient pain medication

**DOI:** 10.1186/1471-2210-11-S2-A8

**Published:** 2011-09-05

**Authors:** Eva-Maria Zebedin, Barbara Zikulnig, Thomas Burkhart, Anna Bucsics

**Affiliations:** 1 Department of Pharmaceutical Affairs, Main Association of Austrian Social Security Institutions, 1030 Vienna, Austria

## Background

In Austria, 98% of the population is covered by (statutory) health insurance. The HVB (Main Association of Austrian Social Security Institutions) publishes the Reimbursement Code (EKO) for outpatient medicines, and if new medicines are submitted for inclusion, these are subjected to pharmacological, medical/therapeutic and health economic evaluation. Providing modern and comprehensive medical care is inevitably and causally linked to a constant rise in health care costs. Treatment of pain has a unique position in every health care system, because it is associated with many diseases of varying severity. Pain patients are confronted both with severe and disabling physical as well as psychological burdens.

## Methods

Data were provided by the internal data warehouse of the HVB, which houses reimbursement data for ambulatory care from 2001 to 2009. In this study we concentrated on the analysis of prescribed DDDs (Defined Daily Doses). Pain medication (as available in the EKO) was grouped according to the WHO defined scheme for treatment of pain (1986) into 3 groups. Aims: First, we aimed to discover changes in prescribing habits. Next, we hoped to learn if and how either greater availability or the restriction of pain medication (e.g. the introduction of generics, the waiving of prescription fees, published severe safety concerns etc,) influence the rate of utilization of pain medication.

## Results

We noticed a difference in the development of the 3 groups: prescribed DDDs of pain medication attributable to WHO stage 1 and 2 show a slight increase from 2003 to 2009 (19% and 22%, respectively). This is followed by a divergent development in the next period: for WHO stage 1, a decrease of prescribed DDDs was noted (−4.6%), whereas there was no marked change for WHO stage 2 (−0.3%). In clear contrast, there was a continuous and significant increase in prescribed DDDs of WHO stage 3 medications, with DDDs almost doubling. To summarize (2003 to 2010): WHO stage 1: +14% (127.5 mil. DDDs), WHO stage 2: +21% (13.8 mil. DDDs), WHO stage 3: +144% (13.4 mil. DDDs).

## Conclusions

So far we can say that the analysis of prescribed DDDs and their allocation into 3 groups – as defined by the WHO scheme for pain treatment – is a useful tool to assess changes in prescribing habits. Next we will try to find out if it can be deduced from our data i) if and how prescribers follow current guidelines, ii) if safety concerns or iii) the introduction of innovative therapies are translated into changes in prescribing habits.

